# Neutrophil Myeloperoxidase Derived Chlorolipid Production During Bacteria Exposure

**DOI:** 10.3389/fimmu.2021.701227

**Published:** 2021-08-13

**Authors:** Kaushalya Amunugama, Grant R. Kolar, David A. Ford

**Affiliations:** ^1^Edward A. Doisy Department of Biochemistry and Molecular Biology, Saint Louis University School of Medicine, St. Louis, MO, United States; ^2^Center for Cardiovascular Research, Saint Louis University School of Medicine, St. Louis, MO, United States; ^3^Department of Pathology, Saint Louis University School of Medicine, St. Louis, MO, United States; ^4^Research Microscopy and Histology Core, Saint Louis University School of Medicine, St. Louis, MO, United States

**Keywords:** neutrophils, 2-chlorofatty acid, 2-chlorofatty aldehyde, plasmalogen, inflammation, *E. coli*, myeloperoxidase

## Abstract

Neutrophils are the most abundant white blood cells recruited to the sites of infection and inflammation. During neutrophil activation, myeloperoxidase (MPO) is released and converts hydrogen peroxide to hypochlorous acid (HOCl). HOCl reacts with plasmalogen phospholipids to liberate 2-chlorofatty aldehyde (2-ClFALD), which is metabolized to 2-chlorofatty acid (2-ClFA). 2-ClFA and 2-ClFALD are linked with inflammatory diseases and induce endothelial dysfunction, neutrophil extracellular trap formation (NETosis) and neutrophil chemotaxis. Here we examine the neutrophil-derived chlorolipid production in the presence of pathogenic *E. coli* strain CFT073 and non-pathogenic *E. coli* strain JM109. Neutrophils cocultured with CFT073 *E. coli* strain and JM109 *E. coli* strain resulted in 2-ClFALD production. 2-ClFA was elevated only in CFT073 coculture. NETosis is more prevalent in CFT073 cocultures with neutrophils compared to JM109 cocultures. 2-ClFA and 2-ClFALD were both shown to have significant bactericidal activity, which is more severe in JM109 *E. coli*. 2-ClFALD metabolic capacity was 1000-fold greater in neutrophils compared to either strain of *E. coli*. MPO inhibition reduced chlorolipid production as well as bacterial killing capacity. These findings indicate the chlorolipid profile is different in response to these two different strains of *E. coli* bacteria.

## Introduction

Neutrophils play vital roles in host defense mechanisms against infections and acute inflammation. They are the initial white blood cells to arrive at sites of infection. Neutrophils kill microorganisms through phagocytosis and the release of antibacterial enzymes. Additionally, neutrophils release neutrophil extracellular traps, (NETs) (1), which may provide an additional mechanism for microbe killing. During neutrophil activation, the primary granules release myeloperoxidase (MPO). MPO uses hydrogen peroxide and chloride to produce hypochlorous acid (HOCl). HOCl has a significant role as an antimicrobial agent and has deleterious effects on host cells. HOCl oxidizes proteins, lipids, and DNA (2–5). Previous studies have shown HOCl targets the vinyl ether bond at the *sn*-1 position of plasmalogen phospholipids liberating 2-chlorofatty aldehyde (2-ClFALD) (6, 7). Plasmalogens are enriched in plasma membranes of neutrophils, endothelial cells, monocytes, smooth muscle cells and cardiac muscles (8–11). 2-ClFALD is relatively short-lived due to its electrophilic nature as well as its metabolism. 2-ClFALD can be oxidized to 2-chlorofatty acid (2-ClFA), can be reduced to 2-chlorofatty alcohol, can form Schiff based adducts with amines and can undergo nucleophilic substitution with glutathione to produce the fatty aldehyde-glutathione adduct (12–15).

Increases in 2-ClFALD and 2-ClFA levels have been demonstrated in both sterile and septic inflammation. 2-ClFALD is elevated in human atherosclerotic lesions as well as infarcted myocardium (16, 17). Endotoxemia leads to elevated plasma levels of 2-ClFA and urinary 2-chloroadipic acid, which is the clearance product of 2-ClFA (18). In human sepsis, increased plasma levels of 2-ClFA associate with acute respiratory distress syndrome (ARDS) and 30-day mortality (19). Similarly, plasma levels of 2-ClFA levels are elevated in experimental septic rats that do not survive (20). Furthermore, in experimental sepsis studies 2-ClFA is elevated in many tissues (20). Other studies have shown blockade of chlorolipid production with MPO inhibitors reduces mesenteric microcirculatory dysfunction (21). In cell studies, 2-ClFALD and 2-ClFA have been shown to elicit endothelial dysfunction, endoplasmic reticulum stress, and apoptosis (22–24). Chlorolipids also are neutrophil chemoattractants and elicit NETosis (7, 25).

Although chlorolipids are produced during sepsis, and neutrophil activation by phorbol esters produces chlorolipids, the production of chlorolipids in response to bacteria has yet to be demonstrated. Additionally, the relative production of chlorolipids by neutrophils in response to different bacteria species or strains could be important mechanistically in the chlorolipid production during sepsis. *E. coli* is one of the common microorganisms causing extraintestinal infections, neonatal sepsis, neonatal meningitis, and bacteremia (26). Unlike most commensal *E. coli* strains enteropathogenic *E. coli* (EPEC) possess virulence factors that allow them to become more invasive. These virulence factors include adhesins, siderophores, toxins, protectins, and invasin that help them to colonize on host mucosal surfaces, injure and invade host cells and escape from host defense mechanisms (27, 28). The CFT073 *E. coli* strain is classified under EPEC strains causing urinary tract infections and sepsis. CFT073 suppresses innate immunity by disrupting the inflammasome that is crucial for pathogen recognition, survival within macrophages, and resistance to phagocyte mediated oxidative stress (29–31).

In this study, we compared chlorolipid production in the presence of *E. coli* CFT073 strain and the *E. coli* JM109 strain. The CFT073 strain generated significant amounts of neutrophil-derived 2-ClFA compared to the JM109 strain. Exogenously added 2-ClFALD was bactericidal to both strains but only the JM109 strain was susceptible to killing by 2-ClFA. 3-Aminotriazole (ATZ) blocked both 2-ClFALD and 2-ClFA production in incubations of neutrophils with either CFT073 or JM109 *E. coli* strains. These are the first studies examining chlorolipid production by human neutrophils elicited by different bacteria and reveal important differences in the production of specific chlorolipids dependent on *E. coli* strain.

## Materials and Methods

### Lipids

2-Chlorohexadecanal and 2-chloropalmitic acid were synthesized and purified as previously described (7, 14). 2-Chlorohexadecanal and 2-chloropalmitic acid were used as representative molecular species of 2-ClFALD and 2-ClFA, respectively, in studies designed to examine the biological roles of these two chlorolipid classes. Hexadecanal and palmitic acid were used to delineate specifics effects of chlorolipids.

### Human Neutrophils

Human neutrophils were isolated from healthy human donors as previously described under Saint Louis University IRB protocol 9952 (7). In brief, healthy human blood was layered on a density gradient in 1:1 volume with blood and centrifuged at 500g for 30 min. The polymorphonuclear cell band was isolated and washed in Hanks’s balanced salt solution (HBSS). Following red cell lysis, the neutrophils were washed twice with HBSS. The isolated neutrophils were suspended in HBSS to prepare the final concentration of 2x10^6^ cell/ml.

### Bacterial Strains and Growth Conditions

CFT073 urosepsis *E. coli* strain and JM109 *E. coli* strain were used in these studies. Bacteria were precultured overnight and subcultured in Luria Bertani (LB) agar broth under shaking condition (250RPM) at 37°C. Once cultures reached the exponential growth phase bacteria number was calculated using a pre-drawn growth curve based on O.D. 600nm spectrophotometric readings. Bacteria were washed and suspended in HBSS to prepare indicated concentrations.

### Neutrophil and Bacteria Cocultures

Neutrophils were cocultured with bacteria in HBSS at 1:10 ratio for indicated time intervals at 37°C. Plasma was not included in these cocultures to minimize the contribution of plasma lipids in analyses. For MPO inhibition studies, neutrophils were preincubated with 10 mM of ATZ for 5 min before the addition of bacteria. Incubations were terminated by the addition of methanol. To quantify chlorolipids that were released into the media *versus* that which was associated with cells, cocultures were centrifuged at 200g for 10 min to prevent neutrophil rupture. Next, the supernatant was further centrifuged at 4700g for 10 min to sediment bacteria and remaining cell debris. The cell pellets following centrifugation were combined to detect cell-associated chlorolipids. Lipids were extracted by the modified Bligh Dyer extraction method as described previously (7, 14, 32). 2-Chloro-[*d_4_*]-hexadecanal and 2-chloro-[*d_4_*]-palmitic acid were used as internal standards as previously described (7, 12, 33, 34).

### Analyses of Chlorinated Lipids

Molecular species of 2-ClFALD were detected following derivatization to their pentafluorobenzyl (PFB) oximes using PFB hydroxylamine. The derivative products were analyzed using GC/MS using selected ion monitoring as previously described (33, 34). Free 2-ClFA was analyzed directly from the lipid extract while total 2-ClFA was measured following base hydrolysis and a modified Dole extraction as previously described (33, 34). 2-Chlorofatty acid molecular species were quantitated following liquid chromatography by selected reaction monitoring using electrospray ionization mass spectrometry (ESI-MS) on a triple quadrupole instrument (Thermo, Altis).

### *E. coli* Killing by Neutrophils

After coculture of neutrophils with *E. coli*, the survival of *E. coli* was assessed by first adding 100U/ml of DNase to eliminate NETs and aggregated cells. For some experiments 2x10^6^/ml neutrophils were pretreated with 10µg/ml of cytochalasin D (cyD) and 10mM ATZ for 15 min and 5 min respectively prior to the addition of *E. coli* and further incubated for 30 min at 37°C. Next, 50µl of the sample was diluted in pH11 water and then incubated at room temperature for 5 min to lyse neutrophils as previously described (35). Sample were subsequently serially diluted in HBSS and plated on LB agar plates. Colony forming units (CFU)/ml were calculated following overnight incubation. The percentage of bacterial survival was calculated by dividing the bacteria number in the coculture by the control bacteria number without neutrophils (the baseline of 100% survival).

### Extracellular DNA Assay

Extracellular DNA (ecDNA) release from neutrophils was assayed as previously described (25). 2x10^6^/ml neutrophils and 20x10^6^/ml *E. coli* were cocultured with 10µM Sytox Green (Invitrogen) and transferred to 96 well black clear bottom plate for incubation at 37°C. ecDNA was detected by fluorescence emission at 523nm by SpectraMax i3 Multi-Mode spectrophotometer. Fluorescence measurements were an average of 21 different regions in a single well to normalize the uneven distribution of ecDNA in the well. ecDNA was expressed as a % of 20 mM saponin-treated neutrophils, which is considered 100% ecDNA.

### NET Isolation and Killing Assay

NET isolation from neutrophils was performed using modifications of a previously described method (36). Briefly, 2x10^6^/ml neutrophils in HBSS were plated on 6 well plates and stimulated with 200nM of phorbol 12-myristate 13-acetate (PMA) in 0.1% ethanol for 4 h at 37°C in the presence of 5% CO_2_. The media was gently aspirated and discarded. The adherent NETs and neutrophils were collected by washing with cold phosphate buffered saline (PBS) and centrifuged at 400g for 10 min at 4°C to remove whole cells and debris. The NET rich supernatant was further centrifuged at 16300g for 10 min at 4°C. DNA in the isolated NET samples were quantified by QuantiFluor dsDNA system (Promega) according to manufacturer’s instructions.

Bacteria killing by NETs was determined by treating 1x10^6^/ml of either CFT073 or JM109 with 50ng/ml of isolated NETs for 30 min at 37°C. Following incubation, 100U/ml of DNase was added for 10 min to break NETs to release the bacteria trapped within NETs. Some experiments were performed with NETs pretreated with 100U/ml DNase. Samples were serially diluted and plated on LB agar plates to determine CFU/ml. Percent bacterial survival was calculated relative to control bacteria without any treatment.

### Phagocytosis Assay

CFT073 and JM109 were labeled with pH sensitive pHrodo deep red as specified by the manufacturer (Invitrogen catalogue no. P35357). Briefly, *E. coli* were harvested from the exponential growth phase and washed twice with the manufacturer-provided washing buffer. The cells were then incubated with pHrodo deep red labeling reagent for 2h at room temperature in the dark. Following labeling, cold LB media was added to scavenge unreacted dye and the cells were washed with HBSS. Labeling was confirmed by exposing the labeled bacteria to acidic pH range and measuring fluorescence intensity and the bacteria viability was also checked by plating on LB agar plates (data not shown).

The phagocytosis assay was performed with slight modifications of a previously described method (37). 5x10^5^/ml of neutrophils and 50x10^5^/ml of pHrodo deep red labeled CFT073 and JM109 were mixed together at Neu: *E. coli* ratio of 1:10 in 96-well black clear bottom plates for the incubation at 37°C. The fluorescence emission was measured at 655nm by SpectraMax i3 Multi-Mode spectrophotometer at given time intervals. Some experiments were performed with neutrophils pretreated with 10 µg/ml of cyD for 15 min before incubation with pHrodo deep red labeled *E. coli*. Net phagocytosis was calculated by subtracting the fluorescence intensity of the *E. coli* only wells (negative control without neutrophils) from the cocultured wells and expressed as a percentage of maximum relative fluorescence units (max RFU).

### Immunofluorescence of NETs

Neutrophils (2x10^6^/ml) in HBSS on coverslips were incubated at 37°C in the presence of either 20x10^6^/ml CFT073 or JM109 bacteria for either 30 min or 2h. At the end of the incubation, cells were fixed with 4% paraformaldehyde for 15 min. Following PBS washing, cells were blocked and permeabilized with 0.5% bovine serum albumin and 1% donkey serum in the presence of 0.05% Triton X-100 for 1 h. Next, cells were incubated with primary antibodies against MPO (1:500) (rabbit monoclonal anti-MPO; Abcam catalog no. ab208670) and *E. coli* (1:200) (goat polyclonal anti-*E. coli*; Abcam catalog no. ab13627) in blocking buffer for 1h at room temperature. Cells were then incubated with secondary antibodies of donkey anti-rabbit Alexa Fluor 594 (1:300) (Jackson ImmunoResearch; catalog no.711-585-152) and donkey anti-goat Alexa Fluor 488 (1:300) (Jackson ImmunoResearch; catalog no.705-545-003) and DAPI (1:2500) (Sigma-Aldrich) for 1 h at room temperature. Slides were mounted with prolong gold antifade reagent. Fifteen contiguous image tiles were captured at 100x (1.40 NA) on a Leica SP8 TCS STED 3X instrument equipped with HyD detectors at full axial depth (0.15 µm increments) of DAPI signal before stitching with Huygens Professional software (SVI, Netherlands). Images were deconvolved in all three channels using Huygens Professional using built in optical parameters and suggested settings. For visualization, 3D reconstructions of image stacks were displayed using built in ray tracing algorithms in Huygens Professional. In all cases, capture settings and visualization thresholds were maintained across groups.

### Lipid Treatment of *E. coli*

Bacteria 50x10^6^ cells/ml in HBSS were treated with indicated lipid concentrations in 0.1% ethanol (EtOH) for an hour at 37°C. Then serial dilutions of bacteria in each condition were subjected to LB agar plating. Bacterial % survival was calculated by dividing bacteria number following the treatment by vehicle-treated (control) bacteria. To quantify bactericidal activity immediately following 2-ClFALD treatment, Live/Dead BacLight Bacterial Viability kit (Invitrogen) was used according to manufacturer’s instructions.

### Neutrophil, *E. coli* and Endothelial Cell Metabolism of 2-ClFALD

Neutrophils (1x10^6^/ml) and bacteria (50x10^6^/ml) were treated with indicated 2-ClFALD concentrations in 0.1% EtOH in HBSS for 1h at 37°C. EA.hy296 cells (passage 4) were grown to 100% confluency and treated with indicated 2-ClFALD concentrations in 0.1% EtOH in Dulbecco’s Modified Eagle Medium with 2% FBS for 1h. Media and cells were collected for 2-ClFA analyses by liquid chromatography-ESI-MS. For some experiments supernatants of neutrophil-bacteria cocultures were added back to fresh neutrophils or bacteria with or without 2-ClFALD.

### Statistics

Student’s t-test was used to compare two groups while one-way ANOVA with Tukey’s *post hoc* analysis and Dunnett’s *post hoc* test were used to compare three or more multiple comparisons. All data were represented as mean with standard deviation (SD) with averages of 3 biological replicates unless otherwise indicated.

## Results

### Neutrophil Chlorolipid Production in the Presence of CFT073 and JM109 Strains of *E. coli*

**Figure 1:**
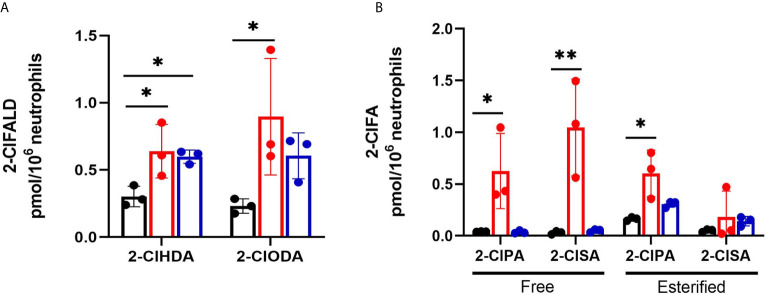
2-ClFALD and 2-ClFA production in cocultures of neutrophils with CFT073 and JM109 *E. coli* strains. 2x10^6^/ml neutrophils were incubated with either no bacteria (control, black), or, CFT073 (red) or JM109 (blue) strains of *E. coli* at a neutrophil: *E. coli* ratio of 1:10 for 30 min at 37°C. 2-ClFALD **(A)** as well as free and esterified 2-ClFA **(B)** were quantified as described in “Materials and Methods”. Data are from three neutrophil donors (biological replicates, 1 female, 2 males). 2-ClHDA, 2-ClODA, 2-ClPA, and 2-ClSA are 2-chlorohexadecanal, 2-chlorooctadecanal, 2-chloropalmitic acid and 2-chlorostearic acid, respectively. Multiple comparisons were performed using one-way ANOVA with Dunnett’s multiple comparison test. Error bars represent ± SD, p-value: ** < 0.01; * < 0.05.

**Figure 2:**
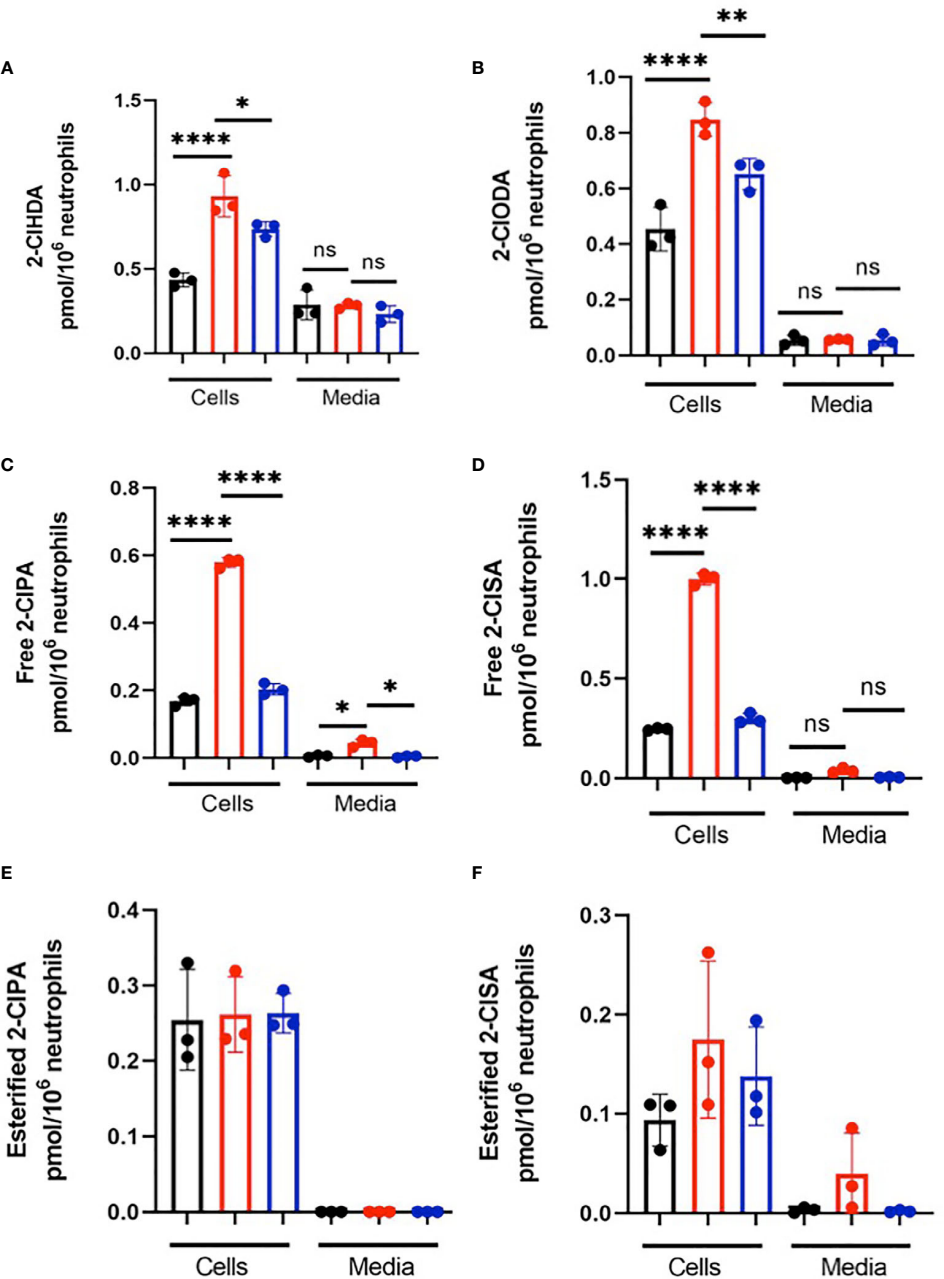
Chlorolipids are cell-associated in neutrophils cocultured with *E. coli.* 2x10^6^/ml neutrophils were incubated with either no bacteria (control, black), or, CFT073 (red) or JM109 (blue) strains of *E. coli* at a neutrophil: *E. coli* ratio of 1:10 for 30 min at 37°C. Cells were pelleted, and 2-ClFALD molecular species **(A**, **B)** and 2-ClFA molecular species **(C–F)** were quantified as described in “Material and Methods”. 2-ClHDA, 2-ClODA, 2-ClPA, and 2-ClSA are 2-chlorohexadecanal, 2-chlorooctadecanal, 2-chloropalmitic acid and 2-chlorostearic acid, respectively. Multiple comparisons were performed using one-way ANOVA with Tukey’s multiple comparison test. Data represent n=3, error bars for ± SD, p-value: ****< 0.0001; **< 0.01; * < 0.05. ns indicates not significant.

Levels of the 2-ClFALD molecular species, 2-chlorohexadecanal and 2-chlorooctadecanal, were significantly elevated in neutrophils exposed to both CFT073 and JM109 *E. coli* strains compared to control neutrophils (**Figure 1A**). The precursor of 2-ClFALD is plasmalogen (6). Plasmalogen is a major lipid in neutrophils, but not *E. coli* (38, 39). In contrast to 2-ClFALD levels, free and esterified 2-ClFA including chloropalmitic acid and 2-chlorostearic acid were increased only in CFT073 co-cultures **(** **Figure 1B** **).** These results were consistent among different neutrophil donors (2-males and 1-female) **(** **Figures 1A, B** **).** Although the trends were consistent for increases in chlorolipids in the presence of *E. coli* strains for each neutrophil biological replicate, we did observe a bimodal distribution of data among the biological replicates. One of the two male biological neutrophil replicates in this study consistently had higher levels of chlorinated lipids compared to the other male replicate and the sole female replicate. Additional studies have shown the majority of chlorolipids (2-ClFALD and 2-ClFA) produced in cocultures of neutrophils with either CFT073 or JM109 *E. coli* strains are cell-associated in comparison to release from cells **(** **Figures 2A–F** **)**.

### Neutrophil Responses to JM 109 and CFT073 *E. coli* Strain

**Figure 3:**
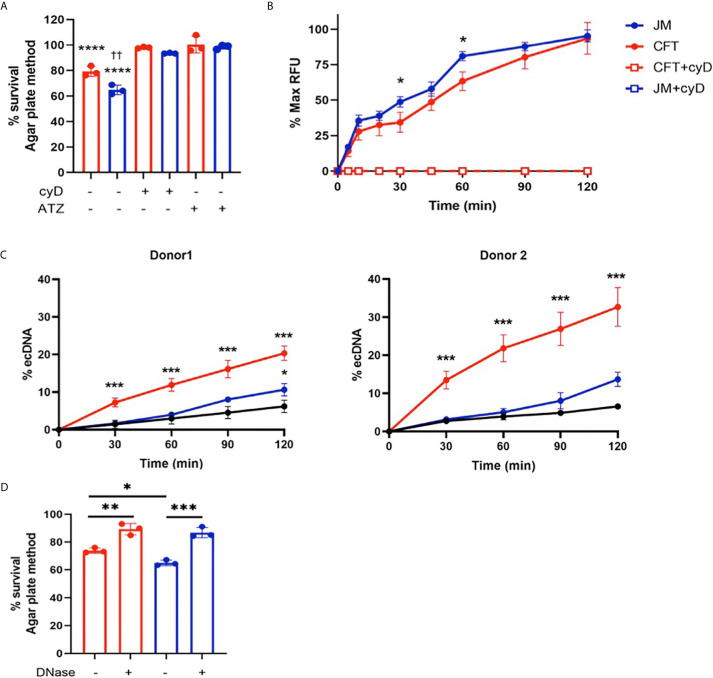
Neutrophil killing mechanisms of *E. coli*. **(A)** 2x10^6^/ml of neutrophils were cocultured with either CFT073 (red) or JM109 (blue) at a ratio of neutrophil: *E.coli* 1:10 for 30min at 37°C. Some coculture experiments were performed with neutrophils pretreated with 10mM ATZ for 5min or 10µg/ml cyD for 15 min. Bacteria survival % was calculated compared to control bacteria. p-value: ****< 0.0001, comparison between each treatment *versus* 100% survival control. p-value: ††<0.01, comparison between neutrophil coculture JM109 and coculture CFT073 cells. **(B)** Neutrophils were cocultured with pH sensitive pHrodo deep red labeled CFT073 or JM109 at neutrophil: *E. coli* ratio of 1:10 as well as in the presence of cyD. Phagocytic response is graphed as % max RFU as described in “Material and methods”. cyD treated CFT073 (red open squares) and JM (blue open squares) data overlap in the graph. **(C)** ecDNA % was measured in the co-cultures and control neutrophils (black) in experiments with two different neutrophil donors by the Sytox green assay as described in “Materials and Methods” (mean ± SD, n=3). **(D)** 1x10^6^ cells of either CFT073 or JM109 strains were incubated for 30 min with isolated 50ng/ml of NETs or NETs pretreated with 100U/ml DNase as indicated. Treatment condition with NETs was further incubated with 100U/ml DNase for 10 min prior to plating on LB plates. Bacterial survival (%) was calculated from CFU/ml relative to control bacteria not exposed to NETs. Values represent the mean ± SD for n=3. Statistics were performed using one-way ANOVA with Tukey’s multiple comparison test **(A, C, D)** and unpaired t-test for neutrophil coculture CFT073 *versus* JM109 in **(B)** p-value; ****< 0.0001; ***< 0.001; **< 0.01; * < 0.05.

**Figure 4:**
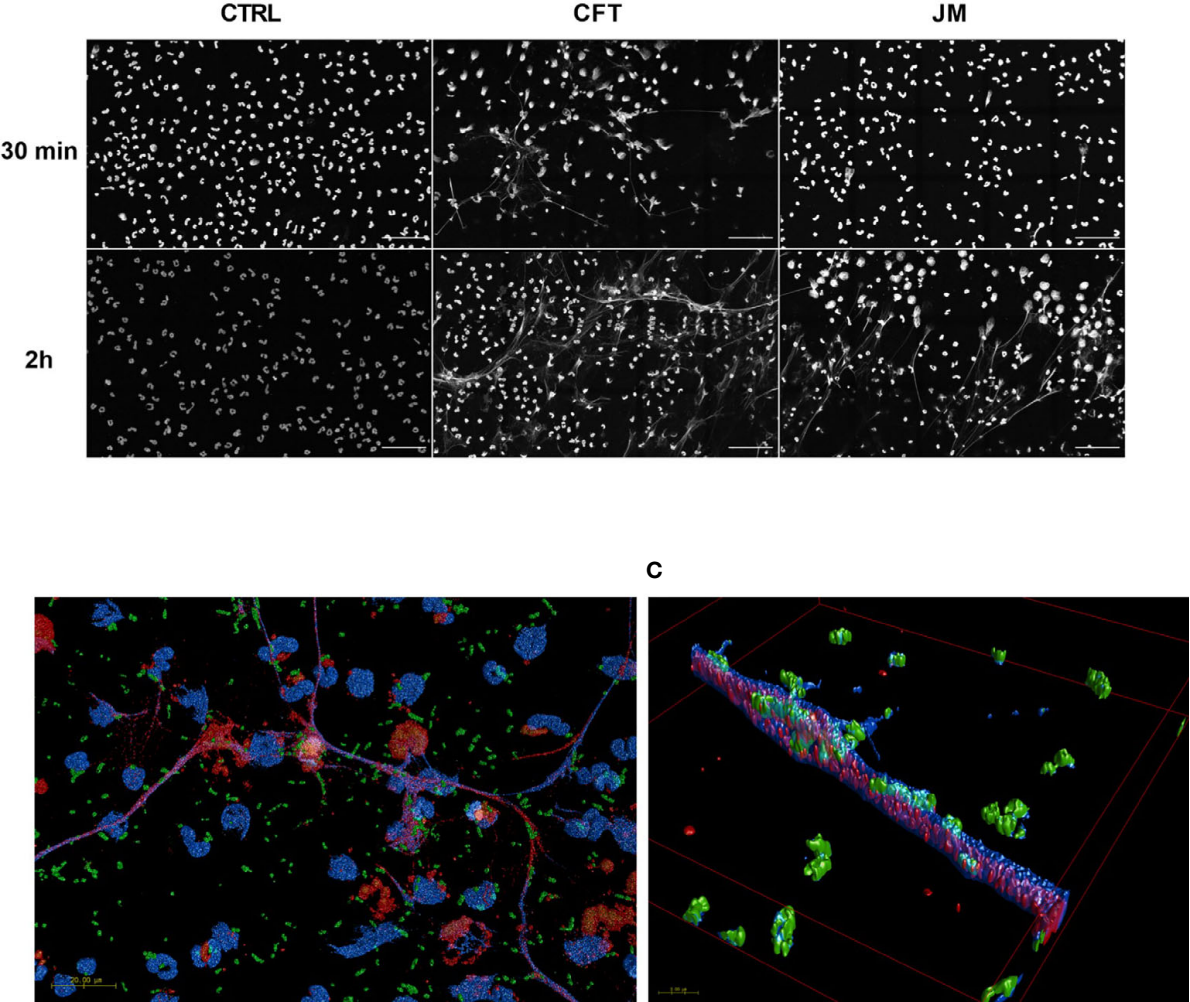
Coculture conditions induce NETosis. 2x10^6^/ml of neutrophils were seeded on coverslips and co-incubated with 20x10^6^/ml CFT073 or JM109 or without bacteria for the indicated time durations at 37°C. Following incubation cells were fixed, permeabilized and stained with immunofluorescence for DNA with DAPI (blue), MPO (red) and *E. coli* (green) as mentioned in materials and methods. **(A)** Large field representation with 15 panels in gray scale using blue channel to show the extent of the NET formation. Scale bar is 50µm. **(B)** 3D representations of confocal data of fifteen 100x tiles of CFT073 cocultures at 30 min. Scale bar 20 µm. **(C)** A zoomed in area on a net **(B)**. The overlap between DAPI (which is made slightly transparent to visualize internal co-localization) and bacteria are seen as a teal color. Overlap between MPO and DAPI appears purple.

Since 2-ClFALD metabolism to 2-ClFA was reduced in neutrophil coculture with JM109 strain compared to CFT073 strain, we next examined other differences in neutrophil responses to these two strains. First, we examined CFT073 and JM109 survival from neutrophil killing. Similar to studies of others (40), data shown in **Figure 3A** demonstrate the JM109 strain is modestly more vulnerable to neutrophil killing mechanisms compared to CFT073 strain. Inhibiting reactive oxygen species production with ATZ as well as phagocytosis with cyD prevented significant neutrophil killing of both *E. coli* strains **(** **Figure 3A** **).** Although others have shown significant non-oxidative killing of *E. coli* (41, 42), it should be noted that conditions employed in the present studies did not include plasma for opsonization. Plasma was omitted to reduce the impact of plasma lipids on lipid analyses during coculture. Phagocytosis of the JM109 strain was marginally greater over time in comparison to phagocytosis of the CFT073 strain (**Figure 3B**). Since 2-ClFA levels are increased in CFT073 cocultures with neutrophils and since we previously observed that 2-ClFA can stimulate NETosis (25), we next investigated NETosis in cocultures by measuring ecDNA. ecDNA formation was significantly increased in CFT073 cocultures with neutrophils at 30 min and increased further over time. In contrast, JM109 cocultures with neutrophils resulted in significant ecDNA only following 2h of coculture **(** **Figure 3C** **)**. Confocal images shown in **Figure 4A** demonstrate the extensive network of NETs formed in cocultures with CFT073 and JM109 *E. coli* strains following 2h of coculture with modest NET formation at 30 min in CFT073 cocultures. Bacteria were trapped in the NETs and MPO was colocalized with NETs (**Figures 4B, C** **)**. Although data shown in **Figures 3A, B** demonstrated phagocytosis is likely the major mediator of JM109 death, we also evaluated the ability of NETs to reduce survival of CFT073 and JM109 *E. coli* strains. NETs were isolated following PMA stimulation, and bacteria were exposed to these NETs for 30 min resulting in reduced viability of CFT073 by 25% and JM109 by 35% (**Figure 3D**). The effect of NETs on *E. coli* survival was reversed by DNase pretreatment.

### Bactericidal Activity of Chlorolipids

**Figure 5:**
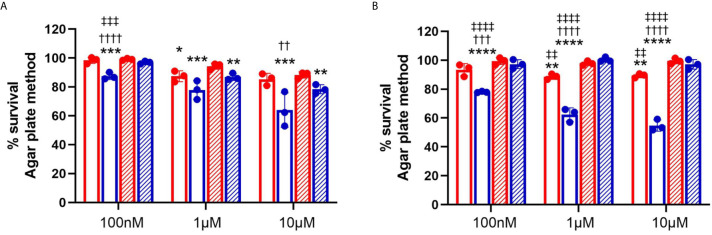
Bactericidal activity of chlorolipids. 50x10^6^/ml of CFT073 (red) and JM109 (blue) cells were incubated with indicated concentrations of either 2-chlorohexadecanal (clear bars, **A**), hexadecanal (hatched bars, **A**), 2-chloropalmitic acid (clear bars, **B**), or palmitic acid (hatched bars, **B**) for 1hr at 37°C. Bacterial survival (%) was determined by calculating CFU/ml over vehicle control bacteria. Values represent the mean ± SD for n=3. Statistics were performed ANOVA within each concentration tested. p-value: ****< 0.0001; ***< 0.001; **< 0.01; *< 0.05 for comparisons for each lipid compared to control (no lipid addition). ^††††^< 0.0001; ^†††^< 0.001; ^††^< 0.01 for comparisons at each concentration for each lipid between treatments of JM109 and CFT073 cells. ^‡‡‡‡^< 0.0001; ^‡‡‡^< 0.001; ^‡‡^< 0.01 for comparisons at each concentration between chlorolipid and non-chlorolipid treatments.

To further understand the role of chlorolipids in bacteria-neutrophil interactions, we tested whether either 2-ClFALD (2-chlorohexadecanal molecular species) or 2-ClFA (2-chloropalmitic acid molecular species) are bactericidal lipids. JM109 and CFT073 viability was measured with either exogenously-added 2-ClFALD or 2-ClFA. In comparison to JM109, CFT073 is more resistant to chlorolipid-elicited killing by both chlorolipids at all concentrations tested (**Figures 5A, B**). JM109 viability to both 2-ClFALD or 2-ClFA decreases in a concentration dependent manner. Moreover, JM109 is more susceptible to 2-ClFA (~50% survival at 10µM) compared to 2-ClFALD (~70% survival at 10µM) (**Figures 5A, B** **)**. In comparison to 2-chloropalmitic acid, palmitic acid did not kill either *E. coli* strains at any given concentration. However, hexadecanal treatment showed killing ability on JM109 at 1 and 10µM levels.

### 2-ClFALD Metabolism in Cocultures and Individual Cells

**Figure 6:**
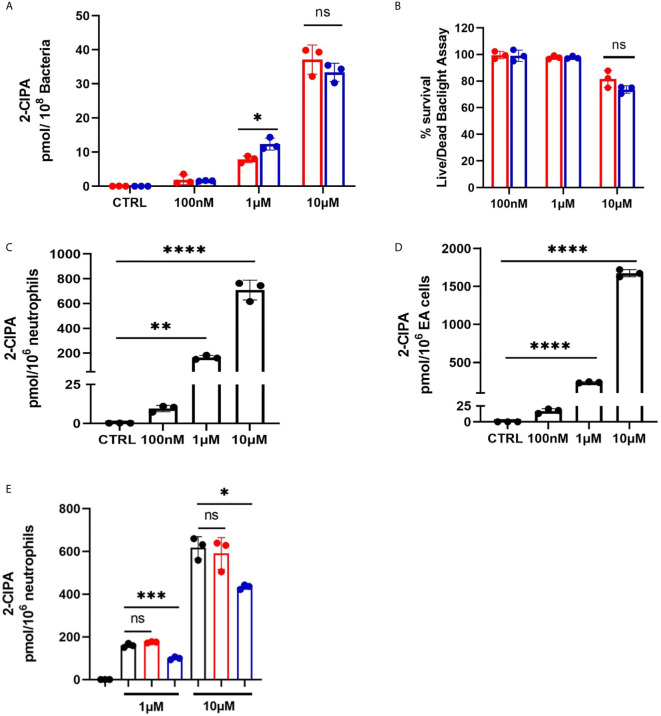
2-ClFALD metabolism in host cells and bacteria. **(A)** Indicated concentrations of the 2-ClFALD molecular species, 2-chlorohexadecanal, in 0.1% EtOH in HBSS media were exogenously provided to 50 x10^6^/ml CFT073 (red) and JM109 (blue) cells for 1h at 37°C. Metabolized free 2-chloropalmitic acid (2-ClPA) was measured as described in “Material and Methods”. **(B)** Following indicated 2-chlorohexadecanal treatments for 1h, CFT073 and JM109 viability was measured using Live/Dead Baclight Bacterial viability kit as described in “Material and Methods”. Percent survival was calculated relative to the control bacteria. **(C)** Neutrophils (1x10^6^/ml) or **(D)** EA hy296 cells (EA) were incubated with indicated concentrations 2-chlorohexadecanal for 1h at 37°C to determine conversion to 2-ClPA. **(E)** Neutrophils were cocultured with either CFT073 (red) or JM109 (blue) with exogenously provided 2-chlorohexadecanal for 30 min and 2-ClPA was measured. Control neutrophils are in black. Statistics were done using unpaired t-test **(A, B)** and one-way ANOVA with Tukey’s multiple comparison test **(C–E)**. Error bars represents ± SD, n=3, p-value: ****< 0.0001; ***< 0.001; **< 0.01; * < 0.05. ns indicates not significant.

**Figure 7:**
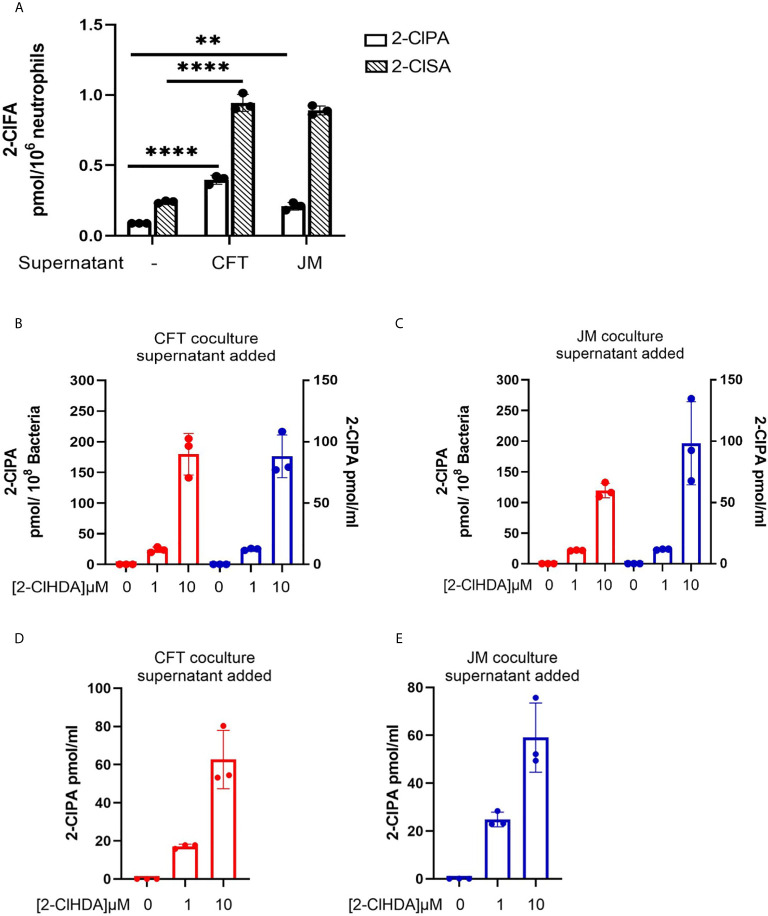
Coculture supernatants amplify 2-ClFA production. 2x10^6^/ml neutrophils were cocultured with 20x10^6^/ml CFT073 or JM109 for 30 min at 37°C and the cells were pelleted. **(A)** The supernatants of CFT073 coculture or JM109 coculture were added to 1x10^6^/ml neutrophils for 1hr at 37°C and free 2-chloropalmitic acid (2-ClPA) and 2-chlorostearic acid (2-ClSA) were measured. **(B, C)** CFT073 coculture supernatant or JM109 coculture supernatant was added to 50x10^6^/ml of CFT073 (red) or JM109 (blue) and incubated with exogenous 2-chlorohexadecanal (2-ClHDA) for 1hr at 37°C and 2-ClPA was measured. **(D, E)** CFT073 or JM109 coculture supernatants were incubated with exogenous 2-ClHDA (0.1% EtOH) for 1 hour at 37°C without bacteria and 2-ClPA was measured. Each condition was performed with n=3 replicates. Statistics were done using one-way ANOVA with Tukey’s multiple comparison test. Error bars represents ± SD, p-value: ****< 0.0001; **< 0.01.

2-ClFALD metabolism to 2-ClFA was examined in CFT073 and JM109 strains as well as in neutrophils. In contrast to disparate metabolism of 2-ClFALD to 2-ClFA in JM109 and CFT073 bacteria strains using endogenously produced 2-ClFALD (**Figure 1**), JM109 and CFT073 metabolized exogenous 2-ClFALD (2-chlorohexadecanal molecular species) nearly equally (**Figure 6A**). Since data in **Figures 5A, B** indicated 2-ClFALD at concentrations as low as 100 nM reduced JM109 survival (as determined by plate assays) we also examined viability (as opposed to ability to proliferate) by an alternate assay using the Live/Dead Baclight viability assay, which indicated both CFT073 and JM109 cells are viable during treatments with 100 nM and 1 μM 2-ClFALD, but both show significant viability loss with 10 µM treatments (**Figure 6B**). Neutrophils metabolized 2-ClFALD to 2-ClFA at a level approximately 1000-fold greater than that observed by either JM109 or CFT073 (**Figure 6C**). Since *in vivo* metabolism of 2-ClFALD during sepsis likely occurs at sites of neutrophil infiltration in the microvasculature, we also examined 2-ClFALD metabolism by EA.hy296 endothelial cells, which was ~two-fold greater than that by neutrophils (**Figure 6D**). To understand whether the coculture environment provides additional factors that modulate 2-ClFALD metabolism to 2-ClFA, we exogenously added 2-ClFALD in the coculture conditions and measured subsequent 2-ClFA production. JM109 coculture condition resulted in significantly lower 2-ClFA production in incubations with either 1 or 10 μM 2-ClFALD compared to CFT073 coculture conditions as well as control neutrophils in the absence of bacteria (**Figure 6E**). In subsequent studies we investigated the possibility that JM109 cocultures with neutrophils release factors that reduce endogenous (by neutrophils) or exogenous 2-ClFALD conversion to 2-ClFA. Data shown in **Figure 7A** show both CFT073 and neutrophil coculture supernatants and JM109 and neutrophil coculture supernatants nearly equally stimulate 2-chlorostearic acid production when applied to neutrophils. There was a modest decrease in 2-chloropalmitic acid production in treatments with JM109 coculture supernatants. Surprisingly, supernatants from both CFT073 and JM109 cocultures with neutrophils resulted in ~2-5-fold accelerated exogenous 2-ClFALD conversion to 2-ClFA when applied to either CFT073 or JM109 strains (**Figures 7B, C** **)** compared to the metabolism of exogenous 2-ClFALD in the absence of coculture supernatant addition (compare to **Figure 6A**). Further studies have shown the increase in 2-ClFALD metabolism to 2-ClFA in the presence of coculture supernatant additions are due to direct metabolic activity present in the supernatant rather than an effect on cellular metabolic activity **(** **Figures 7D, E** **)**.

### ATZ Inhibition of Chlorolipid Production and *E. coli* Rescue From Neutrophil Killing

**Figure 8:**
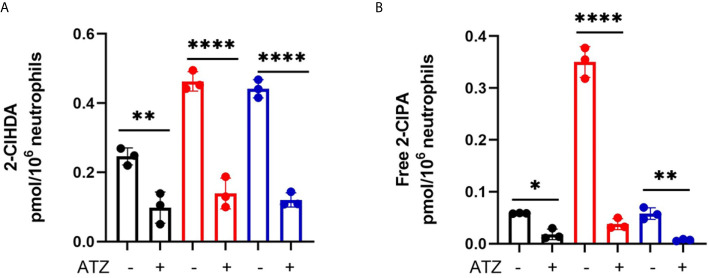
Coculture 2-ClFALD and 2-ClFA production can be inhibited by blocking MPO. 2x10^6^/ml neutrophils were pretreated with 10mM ATZ for 5min and incubated with either no bacteria (control, black), or, CFT073 (red) or JM109 (blue) strains of *E. coli* at a neutrophil: *E. coli* ratio of 1:10 for 30 min at 37°C. 2-Chlorohexadecanal (2-ClHDA, **A**) and 2-chloropalmitic acid (2-ClPA, **B**) were quantified as described in “Material and Methods. Multiple comparisons were performed using one-way ANOVA with Tukey’s multiple comparison test. Data represents n=3, error bars for ± SD, p-value: ****< 0.0001; **< 0.01; *< 0.05.

We have previously shown that MPO inhibition can diminish 2-ClFALD levels in PMA-activated neutrophils (7). Accordingly, we examined the extent of ATZ inhibition of chlorolipid production in cocultures of neutrophils with JM109 and CFT073 *E. coli* strains. Significant reduction of 2-chlorohexadecanal was observed in both bacteria cocultures in the presence of ATZ (**Figure 8A**). Free 2-chloropalmitic acid was also decreased 7-fold with ATZ treatment (**Figure 8B**). Additionally, both JM109 and CFT073 survival in cocultures with neutrophils was improved in the presence of ATZ (**Figure 3A**).

## Discussion

In response to an infection, neutrophils deploy several microbicidal mechanisms against pathogens. Canonical bacterial neutrophil killing includes bacterial phagocytosis, assembly of the NADPH oxidase complex at the phagosome membrane to generate superoxide and subsequently production of HOCl catalyzed by MPO. HOCl is a strong oxidizing agent that reacts with both microbe and host molecules (43–45). HOCl targets plasmalogen phospholipids to generate a family of chlorolipids. 2-ClFALD is the first product of the chlorolipid family, and it is subsequently oxidized to 2-ClFA, which is a stable, relatively long-lived chlorolipid (7, 12, 14). 2-ClFA and 2-ClFALD have profound effects on endothelial cells, monocytes, and neutrophils (7, 19, 22–25). Additionally, although chlorolipids are produced in both human and rodent sepsis (19, 20), the results herein are the first to show the production of 2-ClFALD and 2-ClFA by human neutrophils activated by exposure to bacteria.

Chlorolipid production was investigated in response to both the non-pathogenic K-12 laboratory *E. coli* strain, JM109, and the pathogenic EPEC *E. coli* strain, CFT073. 2-ClFALD, the first product of the chlorinated lipidome was increased in response to human neutrophil exposure to either of these *E. coli* strains. However, 2-ClFA was only increased with neutrophil exposure to the CFT073 *E. coli* strain. Chlorolipids were cell-associated and were not elevated in the cell culture media. Chlorolipid production elicited by JM109 and CFT073 cocultures with neutrophils was inhibited by ATZ. Additionally, these studies are the first to show chlorolipids are bactericidal and, in particular, the JM109 *E. coli* strain is much more susceptible to killing by chlorolipids compared to CFT073. The JM109 *E. coli* strain was very sensitive to killing by 2-ClFA.

The disparate production of 2-ClFA in coculture systems of human neutrophils with JM109 and CFT073 strains of *E. coli* may be due to the differences in the activation of mechanisms involved in neutrophil bacterial killing. Compared to JM109, the CFT073 strain possesses multiple virulence factors. CFT073 harbor pathogenic islands that carry clusters of virulence genes such as autotransporters, hemolysin that are crucial for pathogen survival, invasion, and colonization of human cells (28). In contrast, JM109 strain is devoid of all known virulence factors. Previous studies have also shown that CFT073 is more resistant to neutrophil killing and they are resistant to reactive oxygen species and oxidative stress (30, 40). EPEC strains have also exhibited intracellular survival in human neutrophils (46). Our studies also show the CFT073 modestly survives coculture with neutrophils better than cocultures of JM109. Additionally, considering the known differences in CFT073 and JM109 in resisting oxidative stress it is not surprising that CFT073 is more resilient to survival compared to JM109 when challenged with chlorinated lipids. Furthermore, the disparate resistance to oxidative stress may have a direct effect on 2-ClFALD conversion to 2-ClFA, which is dependent on NAD^+^ (47).

*E. coli* killing and phagocytosis by neutrophils was suboptimal in the present studies in comparison to those of others (41, 42) since *E. coli* were not opsonized. The major goal of these studies was to examine changes in chlorolipids which would be complicated by the addition of chlorolipids and other lipids present in plasma. Under the conditions employed in this study, cyD treatment of neutrophils led to reduced killing of both strains of *E. coli*, which indicated phagocytosis mediates the bacterial killing process under the conditions employed in these studies. Phagocytosis, based on cyD inhibition of bacteria killing, appeared to be the predominant mechanism for killing in the coculture assays. Phagocytosis was also assessed using pHrodo-labeled *E. coli*, which was also cyD-sensitive. Although the pHrodo technique used in this study has been used by others (e.g., 48) to measure phagocytosis by neutrophils there is concern regarding the pHrodo assay in neutrophils based on studies focusing on the pH of the phagosome (49). Using pH-sensitive SNARF-1 labeled dead *C. albicans* the neutrophil phagosome was found to be alkaline for up to 30 min. Additionally, our studies show that in the absence of opsonization, bacteria killing was inhibited by ATZ. This contrast to the non-oxidative dependent killing of opsonized bacteria (41, 42). Based on the disparate requirement of oxidative killing of opsonized and non-opsonized bacteria it will be interesting to evaluate chlorolipid production in response to opsonized bacteria in future studies.

We also examined NETosis in cocultures. We previously found 2-ClFA can elicit NETosis (25). Interestingly, there was a significant increase in ecDNA formation within 30 min in CFT073 cocultures indicating NETosis activation. In comparison, NETosis in JM109 cocultures was delayed. Thus, it is possible that the disparate increase in 2-ClFA levels in CFT073 coculture compared to JM109 coculture may be responsible for the observed NETosis at 30 min in CFT073 cocultures compared to JM109 cocultures. Immunofluorescence images also showed bacterial trapping in NETs. Furthermore, NETs isolated from PMA-stimulated neutrophils had bactericidal activity toward both CFT073 and JM109. This shows NETs can kill these strains, but it should be appreciated that these studies were performed with NETs produced and isolated from PMA-stimulated neutrophils. While these findings are consistent with the concept that NETs prevent microbial dissemination by physically trapping bacteria and/or killing bacteria (50), data with cyD indicate *E. coli* killing in the absence of opsonization is by neutrophil phagocytosis. Although, the role of NETs in bacterial killing is controversial (51), the production of NETs by the uroseptic *E. coli* strain, CFT073, may indicate other roles of NETs in sepsis including detrimental effects on the host such as influencing thrombus formation and disseminated intravascular coagulation (52).

This is the first study to show the bactericidal activity of 2-ClFA and 2-ClFALD. Moreover, the antibacterial effect of chlorolipids is divergent for the two *E.coli* strains examined. It has previously been shown that saturated and unsaturated fatty acids have bactericidal properties, and the antibacterial activity varies with the lipid species and the microorganism strain (53–55). Antibacterial activity of palmitic acid with *E.coli* has previously been shown at concentrations 12-24 higher than the highest concentrations tested in this study (i.e., 10 μM) (56). In comparison, the present studies show 2-chloropalmitic acid toxicity at concentrations as low as 100 nM in the JM109 strain of *E. coli.* It is possible that the α-carbon chlorine is reactive with nucleophiles in *E. coli* leading to the antibacterial activity. We have previously shown 2-ClFALD reacts with glutathione leading to a fatty aldehyde-glutathione adduct (13), and it is predicted that similar reactivity of 2-chloropalmitic acid or its acyl CoA derivative with nucleophiles could occur. Such targeting in *E. coli* needs to be further investigated.

To have a comparative perspective of 2-ClFALD metabolism by cells encountering 2-ClFALD *in vivo*, we examined the metabolism of exogenous 2-ClFALD in endothelial cells, neutrophils and bacteria. Comparisons based on cell number indicated endothelial cells metabolized 2-ClFALD to 2-ClFA about 2-fold greater than neutrophils, and neutrophil 2-ClFALD metabolism was over 1000-fold greater than that of either JM109 or CFT073 *E. coli* strains. Thus, it is likely that *in vivo* metabolism of 2-ClFALD is mediated predominantly by host cells including neutrophils and endothelial cells compared to *E. coli*. Also, an intriguing discovery was that coculture media from incubations of neutrophils with either strain of *E. coli* has activity capable of converting 2-ClFALD to 2-ClFA. The most logical explanation of these findings is that this activity is a result of neutrophil lysis. Future studies will further examine these properties, which might have a significant role in extracellular production of 2-ClFA.

We previously showed fatty aldehyde dehydrogenase mediates the oxidation of 2-ClFALD to 2-ClFA (12). Interestingly, in the present studies we found endogenous 2-ClFALD conversion to 2-ClFA was attenuated in cocultures with JM109 in comparison to cocultures with CFT073. Similarly, exogenous 2-ClFALD conversion to 2-ClFA was reduced in JM109 cocultures with neutrophils compared to CFT073 cocultures (**Figure 6E**). Based on the relative conversion of exogenous 2-ClFALD to 2-ClFA in *E. coli* compared to neutrophils it seems likely that the majority of the metabolism in coculture is mediated by neutrophil fatty aldehyde dehydrogenase. It is possible that factors are released from JM109 cocultures inhibit 2-ClFALD metabolism since media from JM109 cocultures with neutrophils applied to CFT073 cocultures with neutrophils slightly reduced exogenous 2-ClFALD metabolism while the opposite crossover experiment did not alter exogenous 2-ClFALD metabolism by JM109 cocultures with neutrophils. We speculate that the increased neutrophil killing of JM109 compared to CFT073 has a role in the difference in metabolism of endogenous 2-ClFALD to 2-ClFA. It is also possible that JM109 compared to CFT073 has a greater propensity to react 2-ClFALD with nucleophiles. Identifying these potential targets may provide additional insights into differences in neutrophil responses to specific bacteria.

Increased plasma 2-chlorofatty acid levels associate with ARDS-caused mortality in human sepsis (19). Although the origin of elevations in 2-chlorofatty acid in septic humans is likely due to neutrophil activation in response to bacteria, until now the direct production of 2-chlorofatty acid and 2-chlorofatty aldehyde in response to bacteria has not been shown. By comparing neutrophil responses to JM109 and CFT073 strains of *E. coli* we observed that while both strains led to 2-chlorofatty aldehyde, only the pathogenic uroseptic strain CFT073 produced significant amounts of 2-ClFA. Since 2-ClFA is associated with poor outcomes in sepsis and elicits potentially deleterious NET formation it will be important to further understand the mechanisms responsible for the disparate accumulation of 2-ClFA in neutrophils exposed to JM109 and CFT073 as well as the disparate sensitivity of JM109 cells to bactericidal effects of 2-ClFA.

## Data Availability Statement

The original contributions presented in the study are included in the article/supplementary material. Further inquiries can be directed to the corresponding author.

## Ethics Statement

The studies involving human participants were reviewed and approved by Saint Louis University Institutional Review Board. The patients/participants provided their written informed consent to participate in this study.

## Author Contributions

KA performed all experiments, analyzed all data, prepared first draft, and contributed to final manuscript preparation. GK performed image analysis of NETs. DF was responsible for oversight of all aspects of studies, manuscript preparation, and final manuscript. All authors contributed to the article and approved the submitted version.

## Funding

This study was supported (in part) by research funding from the National Institutes of Health R01 GM-115553, R01 GM129508 and S10OD025246 to DF. The content is solely the responsibility of the authors and does not necessarily represent the official views of the National Institutes of Health.

## Conflict of Interest

The authors declare that the research was conducted in the absence of any commercial or financial relationships that could be construed as a potential conflict of interest.

## Publisher’s Note

All claims expressed in this article are solely those of the authors and do not necessarily represent those of their affiliated organizations, or those of the publisher, the editors and the reviewers. Any product that may be evaluated in this article, or claim that may be made by its manufacturer, is not guaranteed or endorsed by the publisher.

## Abbreviations

2-ClFA, 2-chlorofatty acid; 2-ClFALD, 2-chlorofatty aldehyde; ATZ, 3-amino-1,2,4-triazole; ARDS, acute respiratory distress syndrome; CFU, colony forming units; cyD, cytochalasin D; ESI-MS, electrospray ionization mass spectrometry; EPEC, enteropathogenic *E. coli*; EtOH, ethanol; ecDNA, extracellular DNA; HBSS, Hank’s buffered salt solution; HOCl, hypochlorous acid; LB, Luria Bertani; max RFU, maximum relative fluorescence units; MPO, myeloperoxidase; NETs, neutrophil extracellular traps; PFB, pentafluorobenzyl; PMA, phorbol 12-myristate 13-acetate; PBS, phosphate buffered saline; and SD, standard deviation.
